# Detecting long‐term changes in stomatal conductance: challenges and opportunities of tree‐ring δ^18^O proxy

**DOI:** 10.1111/nph.18430

**Published:** 2022-10-06

**Authors:** Rossella Guerrieri, Soumaya Belmecheri, Heidi Asbjornsen, Jingfeng Xiao, David Y. Hollinger, Kenneth Clark, Katie Jennings, Thomas E. Kolb, J. William Munger, Andrew D. Richardson, Scott V. Ollinger

**Affiliations:** ^1^ Department of Agricultural and Food Sciences Alma Mater Studiorum–University of Bologna 4127 Bologna Italy; ^2^ Laboratory of Tree Ring Research, University of Arizona Tucson AZ 85721 USA; ^3^ Earth Systems Research Center University of New Hampshire Durham NH 03824 USA; ^4^ Northern Research Station, US Department of Agriculture Forest Service Durham NH 03824 USA; ^5^ Silas Little Experimental Forest, Northern Research Station, US Department of Agriculture Forest Service New Lisbon NJ 08064 USA; ^6^ School of Forestry Northern Arizona University Flagstaff AZ 86011 USA; ^7^ School of Engineering and Applied Sciences Harvard University Cambridge MA 02138 USA; ^8^ School of Informatics, Computing, and Cyber Systems Northern Arizona University Flagstaff AZ 86011 USA; ^9^ Center for Ecosystem Science and Society Northern Arizona University Flagstaff AZ 86011 USA

**Keywords:** AmeriFlux, dual isotope approach, Péclet effect, precipitation, stable oxygen isotopes, stomatal conductance, tree rings, vapour pressure deficit (VPD)

## A reply to Lin *et al*. (2022) ‘Do changes in tree‐ring δ^18^O indicate changes in stomatal conductance?’

There is growing scientific consensus that rising atmospheric CO_2_ concentrations have caused an increase in forest water‐use efficiency (WUE), but the magnitude of this effect and the relative roles of enhanced photosynthesis vs reduced stomatal conductance (*g*_s_) are still under debate (Walker *et al*., 2020). In Guerrieri *et al*. (2019) we addressed this by combining 30 years of tree‐ring stable carbon (δ^13^C) and oxygen (δ^18^O) isotope data with a water–carbon optimality model for 12 tree species at eight sites within the AmeriFlux network in the US. The results suggested that enhanced photosynthesis has been widespread, while reduced *g*_s_ was more common at sites experiencing frequent water limitation. In this issue of *New Phytologist*, Lin *et al*. (2022) challenges these findings based on three criticisms pertaining to the use and interpretation of δ^18^O data. While the physiological interpretation of δ^18^O in tree rings is indeed complex, it remains the only proxy – in conjunction with δ^13^C – that can be used to reconstruct mechanisms through which WUE changes in response to global change drivers, including atmospheric CO_2_. Here, we address the criticisms raised by Lin *et al*. (2022), with the aim of moving the discussion forward and hence encouraging the use of tree‐ring δ^18^O as a proxy to constrain plant and forest ecophysiology and the response to environmental changes beyond climate variability.

**Criticism 1**: Lin *et al*. (2022) questioned the approach employed in Guerrieri *et al*. (2019) to estimate temporal changes in δ^18^O in precipitation (δ^18^O_P_), which relies on equations presented in Barbour *et al*. (2001) (‘B01 model’ hereafter) to assess spatial δ^18^O_P_. In particular, Guerrieri *et al*. (2019) used temperature and precipitation (both as annual or growing season means) and elevation as predictors. They considered eight sites across North America (most of the sites were in Canada, and thus did not overlap with the sites studied in Guerrieri *et al*., 2019) where δ^18^O_P_ values were available from the global network of isotopes in precipitation (GNIP) for the same time window we considered in Guerrieri *et al*. (2019). The nonsignificant relationship between estimated and measured δ^18^O_P_ found at those sites led the authors to conclude that the B01 model may not be adequate for the estimation of the isotopic signature of precipitation, and that this would, therefore, likely be the case for our sites. We acknowledge that the time for space substitution of the B01 model bears uncertainties that can limit the interpretation of our results. Yet, in the absence of δ^18^O_P_ observations, such an approach is valuable, and we applied it carefully, and in the limited context of our study sites: inferring the directionality of the trend in the transpiration (E)–*g*_s_ relationship. In Guerrieri *et al*. (2019), the δ^18^O_P_ estimates were validated with 2 years of δ^18^O measurements in soil water (δ^18^O_sw_) at two sites with contrasting climates, with the assumption that δ^18^O_sw_ will reflect δ^18^O_P_. As shown in supporting information fig. S11 (Guerrieri *et al*., 2019), the estimates of δ^18^O_P_ were in good agreement with measured δ^18^O_sw_, particularly at the site in Florida, where precipitation patterns may be more complex compared to those of other northeastern US sites, due to the humid subtropical climate. Moreover, we know that measurements made in the first 10 cm of the soil may not accurately reflect δ^18^O_P_ due to the effect of evaporation, thus leading to a deviation between modelled δ^18^O_P_ and measured δ^18^O_sw_; but this did not seem to particularly affect the site in Florida, which had sandy soil and a higher air temperature than the Harvard Forest in northeastern US. The B01 model may not provide a robust estimate of the absolute or interannual variability of δ^18^O_P_ values; however, temperature and precipitation remain two of the most important factors that affect δ^18^O_P_, and these factors provide reliable predictions of global δ^18^O_P_ such as those used in isoscapes (Bowen, 2010). More sophisticated approaches were recently proposed to estimate spatial and temporal changes in precipitation δ^18^O (Terzer *et al*., 2013; Allen *et al*., 2018 at global scale; Nelson *et al*., 2021 for Europe), which will greatly contribute to reducing the uncertainties in source water δ^18^O estimates. When δ^18^O_P_ data are not available, estimating δ^18^O_P_ variations following the B01 model remains a valuable and more simple approach to accounting for the variation in source water δ^18^O in the temporal trend of tree ring δ^18^O, and to improving the detection of tree physiological signals, when the focus is the long‐term pattern (rather than intra‐annual) reconstruction of source water δ^18^O. At most of the sites considered by Lin *et al*. (2022) (see their fig. 1), the estimate from the B01 model and the observed δ^18^O_P_ values showed a consistent directionality of trend (given the positive Pearson coefficient for all but two sites), despite the statistical significance of the correlations. Indeed, values from GNIP usually derive from monthly precipitation water (including snow) collection and therefore have a high temporal resolution which can substantially differ from (smoothed) estimates based on integrated periods such as mean growing season or mean annual temperature and precipitation. A critical point regarding δ^18^O_P_ concerns the different water sources trees can access, that is, different origins (seasonal) and soil depths (Brinkmann *et al*., 2018; Allen *et al*., 2019; Goldsmith *et al*., 2022). We addressed this, though only at two sites (with contrasting climates) where data on soil and xylem water δ^18^O were available (fig. S9 in Guerrieri *et al*., 2019). The estimated δ^18^O_P_ fell within the confidence interval of both measured soil and xylem δ^18^O values. This is aligned with other observations (depending on site, soil type, rooting depth and tree species) that xylem water is similar to soil water (seldom the top soil δ^18^O, e.g. Treydte *et al*., 2014). The uncertainties in the leaf water ^18^O enrichment (Δ^18^O_LW_) estimates that Lin *et al*. (2022) attributed to the estimate of δ^18^O_P_ (obtained from the B01 model, fig. S9 in Guerrieri *et al*., 2019) could be related to the fact that measurements for given days were compared with estimates integrated over the growing season. The absolute values of estimated and measured Δ^18^O_LW_ are not expected to match, given that the measured Δ^18^O_LW_ is based on sample collections from 2–3 d and from two trees (as detailed in the supporting information for Guerrieri *et al*., 2019). Despite that, our estimates fell within the confidence interval of the measured Δ^18^O_LW_, which gives us confidence that our estimates could capture the temporal dynamics of leaf water enrichment.

**Criticism 2**: The second point raised by Lin *et al*. (2022) was related to the interpretation of Δ^18^O_LW_ (as calculated from tree‐ring δ^18^O and estimated δ^18^O_P_) in terms of qualitative changes in *g*_s_, and underlying assumptions regarding the Péclet effect. Our *g*_s_–Δ^18^O_LW_ interpretation was not carried out *a priori*, but in relation to changes in moisture conditions (vapour pressure deficit (VPD), relative humidity (RH), precipitation, standardised precipitation–evapotranspiration index (SPEI)) and evapotranspiration (ET) as derived from eddy covariance data (fig. S15 in Guerrieri *et al*., 2019). We also considered differences in physiological strategies between plant functional type, physiological metrics derived from δ^13^C (intercellular CO_2_ concentrations, carbon isotope discrimination) and basal area increment. That is, we used the relationships found in previous studies (Barbour *et al*., 2000; Sullivan & Welker, 2006; Grams *et al*., 2007) in a conceptual framework in which environmental and physiological metrics were considered to constrain the physiological interpretation of the estimated Δ^18^O_LW_ in terms of *g*_s_ (Siegwolf *et al*., 2021). Interpreting the *g*_s_–Δ^18^O_LW_ relationship as a function of changes in VPD is physiologically plausible, given the well‐established relationship between *g*_s_ and VPD (Grossiord *et al*., 2020). Under more xeric conditions, *g*_s_ is expected to decrease with increasing VPD (Grossiord *et al*., 2020), which is reflected in a reduced dilution of the enriched (in ^18^O) leaf water with nonenriched xylem water at lower transpiration rates at high VPD (Cernusak *et al*., 2016); thus, the Péclet effect has a smaller influence on the predicted leaf water δ^18^O enrichment when compared to measured δ^18^O values (Belmecheri *et al*., 2018). Under mesic conditions and with no temporal changes in precipitation, RH, VPD or SPEI, it is physiologically plausible to assume that *g*_s_ remains unchanged, given that ET at those sites has not changed (fig. S15 in Guerrieri *et al*., 2019). Other studies considering mesic sites in the northeastern USA showed that with the climate becoming wetter, stomatal limitations on photosynthesis are reduced, despite increasing atmospheric CO_2_ concentrations (Levesque *et al*., 2017; Belmecheri *et al*., 2021, the latter including also some of the sites in Guerrieri *et al*., 2019). The finding that *g*_s_ did not decrease over recent decades is in line with other findings based on different data and approaches (e.g. Long *et al*., 2004; Paschalis *et al*., 2017; Purcell *et al*., 2018; Cernusak *et al*., 2019).

The calculation of Δ^18^O_LW_ from tree‐ring δ^18^O in Guerrieri *et al*. (2019) did not account for changes in the absolute values of the effective path length for water movement through the leaf (L factor). The latter may show hourly variations in relation to changes in RH, and those changes followed a similar pattern across a number of days, but the absolute values measured on different days remained within the same range (see, for instance, fig. 1 in Song *et al*., 2013). Diurnal variations in leaf water ^18^O strongly reflect changes in weather conditions, which can be smoothed in tree‐ring δ^18^O, as the latter records a signal integrated over the growing season (several weeks/months) and in response to climate variations. Most of the studies quantifying values of L have been on seedlings and on a few selected days (e.g. Song *et al*., 2013), and these conditions may not be representative of the conditions that mature trees experience throughout the growing season. Nevertheless, if we use the ‘modelling exercise’ presented in fig. 2 by Lin *et al*. (2022), given that ET, RH and precipitation across our mesic sites did not change over recent decades, we can safely assume a fixed L and therefore that the relationship between estimated Δ^18^O_LW_ and *g*_s_ follows the scenario represented by the light blue line (i.e. a negative relationship), in line with our interpretation. Studies integrating tree ring δ^18^O data into land surface models (Keel *et al*., 2016; Barichivich *et al*., 2021) or using it as a proxy in paleoclimatological estimates in tropical regions (Evans, 2007), generally assume a fixed L‐value; however, this did not prevent the interannual variability of tree ring δ^18^O chronologies from being reproduced. Using a fixed L‐value, as applied in early demonstrations of the Péclet effect (see e.g. Barbour *et al*., 2004), will only affect the mean of the absolute modeled Δ^18^O_LW_ values, but not the relationship between observed and measured Δ^18^O_LW_ or Δ^18^O_LW_ and evaporation rate, as shown in figs 3 and 4 in Barbour *et al*. (2004).

**Criticism 3:** Although RH has an important effect on transpiration and, hence, on plant δ^18^O, we observed no systematic trends in humidity over most of our sites and interpreted this as evidence that the temporal trends in δ^18^O observed at some sites were not caused by trends in RH. Lin *et al*. (2022) challenged this, as well as our interpretation that the lack of a trend in Δ^18^O_LW_ at other sites reflected the lack of a trend in *g*_s_. The authors reasoned that the potential for a high degree of interannual variability in RH and the absence of δ^18^O data for atmospheric water vapour increased the possibility that trends in Δ^18^O_LW_ might have gone undetected. Although our study would indeed have benefited from direct measurements of water vapour δ^18^O, those data (as already discussed for δ^18^O_P_) have hardly been measured over the long‐term. At high RH (which could be the case for the northeastern US sites in Guerrieri *et al*., 2019), back‐diffusion of water vapour inside leaves can further complicate the interpretation of the seasonal dynamics of Δ^18^O_LW_ (Lehmann *et al*., 2018), which was not the goal of Guerrieri *et al*. (2019). Nevertheless, a recent analysis provided strong evidence that RH has a much greater effect than water vapour δ^18^O on Δ^18^O_LW_ (Cernusak *et al*., 2022). As such, Lin *et al*. (2022)’s criticisms are speculative and do not offer counter‐evidence that would cause us to alter our interpretation.

Based on their criticisms, Lin *et al*. (2022) concluded that our interpretations of δ^18^O measurements and their implications for trends in *g*_s_ cannot be accepted with certainty. We fully agree but assert that our interpretation is reasonable and consistent with the evidence we presented in Guerrieri *et al*. (2019) and here. Our goal was to apply a novel approach (integrating tree ring isotopes, ecosystem fluxes and modelling) to probe the mechanisms of rising forest WUE and to offer the most plausible interpretation of the available evidence. We are grateful to Lin *et al*. (2022) for highlighting areas of uncertainty that require future attention and would welcome additional evidence that supports or counters our hypotheses. To enhance the physiological interpretation of tree‐ring δ^18^O, future studies should include *in‐situ* observations of source water δ^18^O (i.e. precipitation and water vapour, which could easily be achieved using existing monitoring networks world‐wide, e.g. Fluxnet, NEON, ICP Forests), and a complete characterization of the δ^18^O_P_ pathway from soil through tree xylem (Barbeta *et al*., 2022) and from water vapour to leaf water (bidirectional diffusion – Lehmann *et al*., 2019; Kagawa, 2022) to tree rings under field conditions (both at intra‐ and inter‐annual resolution). Recent progress in deriving precipitation isotope time series using machine learning or isotope‐enabled general circulation models can help to reduce uncertainties related to source water δ^18^O input (Nelson *et al*., 2021). The interpretation of tree ring δ^18^O, however, should go beyond the instantaneous or daily scale assessment of Δ^18^O_LW_, and be integrated with whole tree transpiration data (e.g. through sapflow measurements, now available at the global scale thanks to Poyatos *et al*. 2020) and plant hydraulic traits underpinning water relations (e.g. Flo *et al*., 2021).

## Author contributions

RG drafted the manuscript with input from SB and SVO. All authors revised and contributed to improving the final version of the manuscript.
